# Open science approaches to COVID-19

**DOI:** 10.12688/f1000research.26084.1

**Published:** 2020-08-25

**Authors:** Edwin G. Tse, Dana M. Klug, Matthew H. Todd

**Affiliations:** 1 Department of Pharmaceutical and Biological Chemistry, University College London School of Pharmacy, London, WC1N 1AX, UK

**Keywords:** Sars-CoV-2, COVID-19, open science, open data, open access, open source

## Abstract

In only a matter of months, the coronavirus disease of 2019 (COVID-19) has spread around the world. The global impact of the disease has caused significant and repeated calls for quick action towards new medicines and vaccines. In response, researchers have adopted open science methods to begin to combat this disease *via* global collaborative efforts. We summarise here some of those initiatives, and have created an updateable list to which others may be added. Though open science has previously been shown as an accelerator of biomedical research, the COVID-19 crisis has made openness seem the logical choice. Will openness persist in the discovery of new medicines, after the crisis has receded?

## Introduction

In late 2019, reports began to emerge from Wuhan, China concerning cases of pneumonia of an unknown origin. Shortly thereafter, Chinese authorities identified this to be a novel type of coronavirus disease (now known as coronavirus disease 2019; COVID-19) caused by Severe Acute Respiratory Syndrome coronavirus 2 (SARS-CoV-2), with the outbreak being declared by the World Health Organization (WHO) as a public health emergency of international concern. Over the next two months, increasing numbers of COVID-19 cases were reported in countries outside China at an alarming rate, prompting the WHO to declare COVID-19 a global pandemic in March 2020 ^1^. To date, according to the WHO COVID-19 Situation Dashboard ^2^, there have been around 18 million cases worldwide, in over 200 countries, resulting in around 690,000 deaths. As COVID-19 is caused by a novel coronavirus, there are no established methods for its treatment, and measures such as social distancing and self-isolation have become crucial to prevent further spread. The urgency to overcome this pandemic is furthered by the devastating effect on the world economy that has been seen as a result of the prolonged implementation of these measures ^3^.

The enormous impact of this disease has resulted in significant activity towards new therapeutics, particularly for the development of a new vaccine. Much of this work is taking place in the private sector, alongside the usual requirement in that sector for secrecy. However, there has in parallel been a significant push for a more open approach because it is understood that openness leads to research acceleration. Broadly speaking, these initiatives can be grouped together as three types: 1) open access (the availability of research publications that are free to access and, often, re-use), 2) open data (the same, but with data) and 3) open source (in which broader community participation is allowed *via* liberal licence terms). There are many well-known and frequently-described advantages of openness (e.g. reduction of duplication of effort, faster communication of important outcomes) that nevertheless compete with a need for secrecy for many researchers, arising from the need for protected intellectual property or a perceived competitive advantage. These motivations for secrecy seem, in a time of crisis, to be lessened, and the increased prevalence of open initiatives relating to COVID-19 research has been striking.

Open science in biomedical research has gained increased traction over the past decade ^4, 5^ from screening projects (e.g., CO-ADD ^6^) and the sharing of physical samples (e.g., SGC probes ^7^, MMV Boxes ^8^) through to fully-fledged drug discovery (e.g., Open Source Malaria ^9^, MycetOS ^10^) and development (e.g., M4K Pharma ^11^) campaigns. For COVID-19, data are being generated and shared (e.g. protein target structures, fragment hits), and initiatives have been created to identify and fast-track candidate compounds into clinical use. Even the lengthy process of drug approval is thought to be something that can be shortened: the urgency of the current situation and the use of open science has opened the possibility of reducing the timeline significantly to as little as 1.5 years ^12^ (though the fastest vaccine to be developed, for the 2014–2016 Ebola virus epidemic, took 5 years) ^13, 14^.

**Figure 1.**
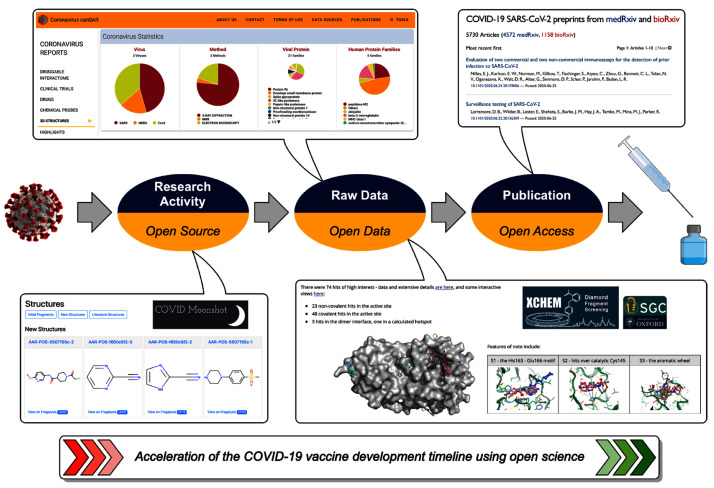
Application of open science for COVID-19 vaccine/treatment development. Representative examples from the development timeline include the COVID Moonshot project, the ICR canSAR tool, the Diamond XChem fragment screen and preprint servers.

This article collates the key open science resources and initiatives currently available for COVID-19 research ( Figure 1). The three previously mentioned categories will be used to group the resources and a brief description of each will be given. This article forms the basis of a “living” collection of open science resources for COVID-19. As more resources become available, anyone may update the repository and discuss those additions.

## Open access

### Preprint servers

An obvious example of open access is the preprint server, such as bioRxiv ^15^ and medRxiv ^16^. To date, there have been more than 7000 COVID-19 focussed articles submitted to these servers ^17^. While this is a valuable resource, the articles are preliminary reports and are yet to be peer-reviewed: the main purpose of preprints is to allow researchers to quickly disseminate their results before official publication. It is noted that this surge in COVID-related studies has led to preprint servers like bioRxiv to implement stricter quality control of submitted articles, resulting in purely computational-based papers no longer being accepted ^18^.

### Journals

Unlike preprint servers, open access journals publish peer-reviewed articles that are freely available for anyone to view without payment. Examples of open access journals featuring COVID-19 collections include the Public Library of Science journals (e.g. *PLoS One*, *PLoS Medicine*, etc.) ^19^, *Nature Communications* ^20 ^and Wellcome Open Research ^21^. Other journals which are not fully open access, such as *JAMA* and *The BMJ*, have made their COVID collections free and open ^22, 23^.

## Open data

### Diamond protease and fragment data

The Diamond Light Source is a synchrotron facility located in the UK that is utilised for a range of scientific areas including the investigation of protein structures and properties. During the coronavirus pandemic, Diamond scientists have used their facility to generate data on protein targets and fragments, all of which have been made publicly available ^24^. Notably, by solving a high-resolution structure (1.39 Å) of the SARS-CoV-2 main protease ^25^, it was possible to perform a screen of multiple fragment libraries to identify the most promising hits for fragment-based drug discovery. This screen resulted in 74 high interest hits and the full results of this screen have been made available to researchers and initiatives such as the PostEra COVID Moonshot project ( *vide infra*). This main protease structure has also been used to screen for covalent probes from a library of electrophile fragments ^26^.

### The Protein Data Bank

The Protein Data Bank (PDB) is an open database containing the 3D structural data of proteins and nucleic acids deposited by researchers from around the world. This database is an important resource for scientific research and many scientific journals now require authors to submit their structural data to the PDB. The PDB are maintaining a collection of a wide range of SARS-CoV-2 structures including the main protease and spike protein/receptors ^27^.

### NCATS OpenData COVID-19

The National Center for Advancing Translational Sciences (NCATS) has focussed its efforts on drug repurposing for COVID-19 by generating datasets created from the screening of SARS-CoV-2-related assays against FDA-approved drugs and anti-infectious agents. Multiple compound collections are actively being screened in eight assays (with more in development) that focus on various stages of the SARS-Cov-2 life cycle in both human and viral targets. The results of the screen, as well as all assay protocols, have been made available online ^28^.

### ICR Coronavirus canSAR

The Institute of Cancer Research (ICR) have developed a tool named canSAR which is a knowledgebase that collates multidisciplinary data and applies machine learning approaches to provide useful predictions for cancer drug discovery. The ICR have repurposed this tool for the current research efforts against coronavirus, allowing people to freely search for information including the druggable interactome, ongoing and completed clinical trials, and lists of active compounds, probes and targets under investigation ^29^.

### Data aggregation initiatives

A number of platforms have been created with the purpose of aggregating and curating openly available data that has been generated during COVID-19 research. The European Commission is working with a number of partners, including the EMBL-EBI, to create a platform that aggregates data ranging from sequencing and expression data to protein structures, drug targets and compounds ^30^. The CORD-19 dataset is a large, machine-readable database intended to facilitate machine learning and data mining approaches to COVID-19 research ^31, 32^. The COVID-19 Molecular Structure and Therapeutics Hub maintains a repository of input files and analysis scripts for molecular simulation and dynamics studies related to COVID-19 ^33^.

## Open source

### PostEra COVID Moonshot

PostEra AI is a for-profit startup company that specialises in integrating molecular design with chemical synthesis. As a result of the coronavirus pandemic, and stemming from the data produced by the Diamond Light Source ( *vide supra*), PostEra have collaborated with academic institutions and industry around the world, while adopting open science principles, to design new inhibitors of the SARS-CoV-2 main protease ^34^. This global collaboration effort allows anyone to suggest new inhibitors based on the initial Diamond fragment hits. Following this, the most attractive compounds will be identified using machine learning algorithms, synthesised by a contract synthesis company and evaluated in inhibition assays (fluorescence and RapidFire mass spectrometry) against the SARS-CoV-2 main protease in labs around the world. Importantly, all stages of this process will be made publicly available. To date, there have been over 12000 unique compounds designed by the community. Over 1300 compounds have been ordered commercially, around 950 compounds have been synthesised, and over 900 compounds have been assayed against SARS-CoV-2.

### JEDI GrandChallenge

The Joint European Disruptive Initiative (JEDI) is a search for breakthrough technologies in the European Union. The GrandChallenge is a three stage campaign for the development of lead compounds against multiple SARS-CoV-2 targets ^35^. Stage 1 is an open competition that focuses on *in silico* screening of compounds against high-resolution protein structures. Teams use simulation approaches (e.g. molecular dynamics, deep learning, docking, etc.) to score libraries of compounds against a chosen target. By comparing multiple different approaches errors can be averaged out and the best compounds for each protein target chosen for progression to the next stage. Stage 2 is an *in vitro* screening stage focussing on identifying the compounds from Stage 1 that provide 99% viral suppression. Teams must provide experimental evidence for this through either selective testing, high-throughput screening or smart combinatorial methods. Stage 3 is the *in vivo* screening stage aimed at finding novel drug combinations. This stage is run independently from the first two stages but lead compounds from those stages may be incorporated in this stage (provided they have been FDA-approved). Following the conclusion of each stage, the top-ranked team will be awarded a cash prize (€250,000 in both Stage 1 and 2, and up to €1,000,000 in Stage 3).

### Sample sharing: MMV COVID Box

The Medicines for Malaria Venture (MMV) is a not-for-profit organisation that brings together the public and private sectors for the discovery and development of new antimalarial medicines. MMV have previously created, and freely distributed, collections of promising candidate compounds in well plates to researchers around the world to enable a more efficient starting point for new drugs (see Malaria Box ^36^, Pathogen Box ^37^ & Pandemic Box ^38^). MMV have now created, and made available on request, the COVID Box, which contains 80 compounds of both marked drugs and compounds in development that possess known or predicted activity against SARS-CoV-2 ^39^. A stipulation of this open research project is that the resulting data generated by researchers using the COVID Box must be shared in the public domain within 2 years of its generation.

### Sample sharing: COVID-19 Protein Portal

The COVID-19 Protein Portal is a UK-based initiative led by Wellcome and UKRI, that provides SARS-CoV-2-related protein reagents for UK scientists to use, free of charge ^40^. These include viral proteins, human proteins and antibodies, all of which are searchable in their online database. All results generated from the use of these reagents will be made publicly available.

### Nextstrain pathogen evolution

Nextstrain is an open source, interactive data visualisation platform that provides “real-time tracking of pathogen evolution” *via* the analysis of sequencing data ^41^. The tools used to achieve this are freely available to use and modify, and have already been used to track the evolution of a range of pathogens including the seasonal flu, Zika virus, and the West Nile virus. In response to the current pandemic, Nextstrain is maintaining a SARS-CoV-2 phylogenetic tree based on the analysis of contributed sequencing data.

### Folding@home

Folding@home (F@H) is a distributed computing project involving multiple research labs and citizen scientists from around the world that focuses on simulating protein dynamics. F@H provides software that enables users to donate unused computing power towards the computational analysis of protein folding ^42^. Thus far, F@H’s COVID-19 projects have focused on simulating the interactions between the SARS-CoV-2 spike protein and the human ACE2 receptor to which it binds. All input files are available through GitHub, which is also where the research outputs will be made openly available ^43^.

### Open Source COVID-19 Research Consortium

The Open Source COVID-19 (OSC19) Drug Discovery program is utilising computer science and biochemistry to enable the rapid screening of existing drug molecules for use against COVID-19 ^44^. Scientists from a range of fields are encouraged to participate, including synthetic chemists to make drug candidates, biochemists and virologists to run assays and donors and volunteers to aid in publicity and fundraising. As an open source project, all research results will be freely available with no intellectual property claims for any discoveries made.

## Other resources

In a similar manner to this article, a number of additional resources have been created to help spread word of the growing list of open science efforts for COVID-19 research. Examples include Initiatives such as Joinup EU ^45^ and SPARC ^46^, which have created hubs of open source research projects and resources. Funding agencies like the UKRI and UKCDR have provided lists of funded COVID-19 research projects to help researchers identify and fill funding gaps ^47, 48^. To further advocate the use of open science, the Open COVID Pledge has aimed to encourage researchers and businesses to make their COVID-related intellectual property freely available by providing the Open COVID Licence ^49^. Finally, the Virus Outbreak Data Network (VODAN) is working to ensure that data related to the COVID outbreak is findable, accessible, interoperable, and reusable ^50^.

## Conclusion

A significant amount of effort has been made to progress COVID-19 research since the beginning of the pandemic. With so many scientific minds working on this problem together, it is clear that conducting scientific research in an open manner can accelerate the research process. The resources and initiatives highlighted in this article demonstrate the benefits of open science approaches and its potential to accelerate research timelines. This article itself will form the basis of an open science “living” resource hosted on a public repository. It is understandable that a global public health crisis causes us to adopt innovations in how we work in the search for an effective solution. After the COVID-19 crisis has faded there will remain many other crises that we face in the search for an effective therapy to alleviate suffering, whether the affected population is a billion people or a single individual in search of a cure. It is hoped that open science will be seen as a “new normal” approach in those crises too.

## Data availability

### Underlying data

No data are associated with this article.
