# Supplementary material for: Open science approaches to COVID-19
Source: F1000Res. 2020 Aug 25;9:1043. [Version 1] doi: 10.12688/f1000research.26084.1 (PMC7590891; doi:10.12688/f1000research.26084.1)
Supplement: Peer review history

# Peer review: Reviewer response for version 1

**DOI:** 10.5256/f1000research.28785.r70312

**Published:** 2020-10-26

**Authors:** Alexander Tropsha

**Affiliations:** 1 Laboratory for Molecular Modeling; UNC Eshelman School of Pharmacy, University of North Carolina at Chapel Hill, Chapel Hill, NC, USA

This is a very timely, excellent manuscript that both highlights and supports open drug discovery research especially for COVID-19 and possible future pandemics. The authors provide a nice overview of different types of open research activities related to the research response to COVID-19.

 There has been indeed an unprecedented level of sharing data and resources since the beginning of the pandemic and it is difficult to cover all of these developments in a short review. I would nevertheless suggest a few additions. Thus, I recommend adding a statement on the availability of computational resources besides Folding@home such as COVID-19 HPC consortium ( https://www.xsede.org/covid19-hpc-consortium).  

 I would also suggest a broader acknowledgment of multiple journals and publishers that agreed to provide open access to all papers on the topic of COVID-19. A possibly complete list is available from the Wellcome Trust (https://wellcome.org/press-release/publishers-make-coronavirus-covid-19-content-freely-available-and-reusable). 

 Finally, I would recommend that the authors expand on the important issue of the quality of research publications. The authors mention that even preprint servers stopped accepting manuscripts with countless purely computationally repurposed drugs against the disease with no experimental confirmation. It would be prudent to cite a recent publication in Nature Biotechnology on the need for automated peer review of the publications tsunami ( https://www.nature.com/articles/s41587-020-0686-x) ^1^ and perhaps, emphasize the importance of rigor, experimental support, and reproducibility of research findings reported in both preprint servers and peer-reviewed publications.

Is the review written in accessible language?

Yes

Are all factual statements correct and adequately supported by citations?

Yes

Are the conclusions drawn appropriate in the context of the current research literature?

Yes

Is the topic of the review discussed comprehensively in the context of the current literature?

Yes

Reviewer Expertise:

Cheminformatics and Computer Aided Drug Discoevry

I confirm that I have read this submission and believe that I have an appropriate level of expertise to confirm that it is of an acceptable scientific standard.

## References

[1] : Artificial intelligence, drug repurposing and peer review. *Nat Biotechnol*.2020;38(10) : 10.1038/s41587-020-0686-x 1127-1131 10.1038/s41587-020-0686-x 32929264

# Peer review: Reviewer response for version 1

**DOI:** 10.5256/f1000research.28785.r70313

**Published:** 2020-09-14

**Authors:** Matthew Hall, Kyle Brimacombe

**Affiliations:** 1 National Center for Advancing Translational Sciences, National Institutes of Health, Rockville, MD, USA

**Comments to the Author**

 The authors have worked to summarize the open science efforts made in the wake of the COVID-19 pandemic. They have collected COVID-19-related open research resources and categorized them as open access, open data, or open source. In summarizing these approaches, the authors seek to highlight the benefits of open science and the promise of accelerated therapeutic development in this unprecedented COVID era.

 The review as a whole is succinct, well-written and easy-to-understand, and presents a clear summary of the accumulated open science resources. The curation work the authors have undertaken is commendable, and given the number of COVID-related initiatives becoming public, it could prove valuable to researchers looking to find an appropriate resource.

 It’s clear the review was meant to summarize the state of open science initiatives for COVID-19 research without offering commentary or analysis on the approaches taken, but this reviewer can’t help but wonder what lessons the scientific community will learn from this first big foray (for many) into open science. E.g. who were the main users of these open science resources? Are there (or should there be) minimum requirements to be considered an “open science” resource? Is it enough to say that data/results will be made public in the future?

 Regarding the list being compiled by the authors, this reviewer understands why Github is a logical home for a living, open source resource, but it may inadvertently limit the audience reached (or require a bit of exploration for users new to the platform). Github will likely be familiar to those in the informatics or developer communities, but to those in basic/bench research, the sight of a Github page may signal that the resource is not meant for them. The authors might consider mirroring the resource somewhere in a format more familiar to bench researchers or perhaps work to direct users to the wiki rather than the code tab, where the sight of a classic README.md file/structure might scare away potential readers who otherwise recognize that as the sign of a wrong turn taken.

 To improve the utility of their lists, the authors might also consider categorizing the resources by subject matter (e.g. structural data vs small molecule screening/activity vs prediction platforms) in addition to classifying by type of open science resource. It’s likely that users may be in search of a particular type of COVID resource/dataset matched to their area of expertise, more so than a particular class of open science platform (open data vs open source). Those in search of the former will need to read through a handful of separate resource write-ups before being able to decide which, if any, are relevant to them.

 The value of this type of resource is highly dependent on how frequently it is updated and curated, so the authors should be commended on their undertaking of this work.

**Minor edit to text:**

 The NCATS OpenData Portal (cited as #28) also has a preprint which goes into greater detail about the mission and design of the site: https://www.biorxiv.org/content/10.1101/2020.06.04.135046v1 ^1^

Is the review written in accessible language?

Yes

Are all factual statements correct and adequately supported by citations?

Yes

Are the conclusions drawn appropriate in the context of the current research literature?

Yes

Is the topic of the review discussed comprehensively in the context of the current literature?

Yes

Reviewer Expertise:

Open data sharing, Translational science, Assay development, Experimental therapeutics, Drug discovery

We confirm that we have read this submission and believe that we have an appropriate level of expertise to confirm that it is of an acceptable scientific standard.

## References

[1] : An OpenData portal to share COVID-19 drug repurposing data in real time. *bioRxiv*.2020; 10.1101/2020.06.04.135046 10.1101/2020.06.04.135046 32511420PMC7276055
